# Identification of foodborne pathogenic bacteria using confocal Raman microspectroscopy and chemometrics

**DOI:** 10.3389/fmicb.2022.874658

**Published:** 2022-11-07

**Authors:** Jin Zhang, Pengya Gao, Yuan Wu, Xiaomei Yan, Changyun Ye, Weili Liang, Meiying Yan, Xuefang Xu, Hong Jiang

**Affiliations:** ^1^Criminal Investigation School, People’s Public Security University of China, Beijing, China; ^2^State Key Laboratory for Infectious Disease Prevention and Control, National Institute for Communicable Diseases Control and Prevention, Chinese Center for Disease Control and Prevention, Beijing, China

**Keywords:** foodborne pathogenic bacteria, confocal Raman microspectroscopy (CRM), pretreatment, chemometrics, classification

## Abstract

Rapid and accurate identification of foodborne pathogenic bacteria is of great importance because they are often responsible for the majority of serious foodborne illnesses. The confocal Raman microspectroscopy (CRM) is a fast and easy-to-use method known for its effectiveness in detecting and identifying microorganisms. This study demonstrates that CRM combined with chemometrics can serve as a rapid, reliable, and efficient method for the detection and identification of foodborne pathogenic bacteria without any laborious pre-treatments. Six important foodborne pathogenic bacteria including *S. flexneri*, *L. monocytogenes*, *V. cholerae*, *S. aureus*, *S. typhimurium*, and *C. botulinum* were investigated with CRM. These pathogenic bacteria can be differentiated based on several characteristic peaks and peak intensity ratio. Principal component analysis (PCA) was used for investigating the difference of various samples and reducing the dimensionality of the dataset. Performances of some classical classifiers were compared for bacterial detection and identification including decision tree (DT), artificial neural network (ANN), and Fisher’s discriminant analysis (FDA). Correct recognition ratio (CRR), area under the receiver operating characteristic curve (ROC), cumulative gains, and lift charts were used to evaluate the performance of models. The impact of different pretreatment methods on the models was explored, and pretreatment methods include Savitzky–Golay algorithm smoothing (SG), standard normal variate (SNV), multivariate scatter correction (MSC), and Savitzky–Golay algorithm 1st Derivative (SG 1st Der). In the DT, ANN, and FDA model, FDA is more robust for overfitting problem and offers the highest accuracy. Most pretreatment methods raised the performance of the models except SNV. The results revealed that CRM coupled with chemometrics offers a powerful tool for the discrimination of foodborne pathogenic bacteria.

## Introduction

The World Health Organization (WHO) survey results indicated that foodborne diseases are increasingly reported as serious public health problems around the world (Wu et al., 2013). Billions of people in the world are at risks of unsafe food (Fung et al., 2018). Millions of people are infected with foodborne diseases every year. It leads to high rates of morbidity and mortality. Meantime, since most of foodborne pathogenic bacteria can survive and even multiply in the harsh environmental conditions, it also presents huge challenges to the production, processing, and storage of food products for the food industry (Giaouris et al., 2015). Foodborne diseases not only are a serious threat to the health of the people, but also cause inestimable loss of property to consumers and food-related industries. It also poses dramatic negative impact to economic growth, political, and social stability of the country (Chen and Alali, 2018; Zhou et al., 2019; Mi et al., 2021). Thus, it also poses greater importance to solve this problem in view of the seriousness and harmfulness of foodborne diseases (Mi et al., 2021).

The most commonly well-known bacterial pathogens in connection with foodborne diseases worldwide include *Shigella*, *Listeria monocytogenes*, *Vibrio cholerae*, *Staphylococcus aureus*, *Salmonella*, and *Clostridium botulinum* (*C. botulinum*) (Chen and Alali, 2018). The constant threats from these bacterial pathogens make rapid and cost-effective detection and discrimination of foodborne pathogenic bacteria a crucial issue for environmental monitoring, food safety, and early diagnosis of diseases (Kant et al., 2018; Yin et al., 2020). Traditional culture-based methods are the common and mature techniques for the detection of bacterial pathogens, and simple operation and low cost are the main reason to make these methods popular, whereas it is a slow process and not to achieve the aim for rapid detection in today’s food industry (Wu et al., 2013). Methods based on immunology include enzyme-linked immunosorbent assay (ELISA) (Vaz-Velho et al., 2000; Ferreira et al., 2001; Hochel et al., 2004), immunomagnetic separation technique (Wang and Slavik, 1999; Taban et al., 2009), and immunofluorescence labeling technique (Yan et al., 2008). ELISA is more common among them, and the advantages of ELISA are fast separation speed, high sensitivity, and specificity for bacterial types and strains, whereas rapid detection using ELISA in the field is impractical due to the requirement of multiple steps, various chemical reagents, and time-consuming incubation (Wu et al., 2013). Molecular biology methods have been extensively adopted for microbial detection and identification in the past few decades (Mi et al., 2021), such as pulsed-field gel electrophoresis (PFGE) (Umeda et al., 2009; Skarin et al., 2010; Anza et al., 2014), amplified fragment length polymorphism (AFLP) (Keto-Timonen et al., 2005), DNA microarray (Artin et al., 2010; Raphael et al., 2010; Vanhomwegen et al., 2013; Ng and Lin, 2014), multilocus sequence typing (MLST) (Macdonald et al., 2011; Olsen et al., 2014), and polymerase chain reaction (PCR) (Szabo et al., 1994; Braconnier et al., 2001; Lindström et al., 2001; Dahlsten et al., 2008; De Medici et al., 2009). These methods can be carried out without time-consuming incubation compared to culture-based methods, but there are some inevitable restrictions limiting their applications. For example, PCR is based on nucleic acid amplification and consequently cannot discriminate nucleic acid amplified from viable and non-viable bacteria (Wu et al., 2013). In summary, many existing detection techniques have great limitations (Mi et al., 2021), and it is necessary to find a rapid and efficient method for rapid multispecies tests (Maquelin et al., 2002; Ho et al., 2019).

Raman spectroscopy has already been recognized as a powerful analytical technique for rapid characterization and detection of bacteria without external labels or tedious preparation. The Raman spectrum deriving from molecular vibrations can be considered as a typical whole-organism fingerprint of the biochemical composition of microorganisms. This vibrational spectrum could show the differences of the molecular compositions in various bacterial pathogens at the molecular level (Maquelin et al., 2002; Lin et al., 2019). Thus, Raman spectra can be used to infer strain-specific physiological, metabolic, and phenotypic states of bacterial cells (Chisanga et al., 2020).

Confocal Raman microscopy (CRM) is a powerful optical spectroscopy technique. It combines Raman spectroscopy with a confocal microscope. It is with advantages such as its fingerprint-identification capability and great sensitivity in aqueous medium (Andrei et al., 2020). It gives the opportunity to identify single bacterial cell in high spectral resolution, combining the power of 3D sample analysis with focused biological component. A laser beam of approximately 1 mm with known wavelength is used to analyze a sample. The scattered radiation and energy shift are measured, and differentiation of species and even strain level is achieved by the acquired chemical characteristic information of the sample (Serrano et al., 2015; Kriem et al., 2020). In biomedicine, CRM has been applied in the discrimination, classification, and diagnosis of pathological conditions, such as various malignancies and tumors. However, few reports have addressed the use of this technique in the detection and discrimination of foodborne pathogenic bacteria.

Since the main biological components are similar in different foodborne pathogenic bacteria, such as nucleic acids, proteins, lipids, and carbohydrates, it always leads to high similarity of Raman spectrum. Thus, it is important to apply chemometrics to spectral data for distinguishing different bacteria species. Statistical approaches include unsupervised techniques and supervised techniques. In the unsupervised techniques, unlabeled datasets are analyzed and clustered without the need for human intervention. Principle component analysis (PCA) is one of the common unsupervised technique for spectral analysis (Kriem et al., 2020). In the supervised techniques, the aim is to classify data or predict outcomes accurately in labeled datasets. Some classical supervised classifiers include decision tree (DT), artificial neural network (ANN), and Fisher’s discriminant analysis (FDA).

In this study, we aim to evaluate and examine the potential of the CRM and chemometrics methods for the detection and classification of six foodborne pathogenic bacteria. We also explored the impact of different pretreatment methods on the models including Savitzky–Golay algorithm smoothing (SG), standard normal variate (SNV), multivariate scatter correction (MSC), and Savitzky–Golay algorithm 1st Derivative (SG 1st Der). PCA was used for investigating the difference of various samples and reducing the dimensionality of the dataset and extracting feature. Performances of classical classifiers were compared for bacterial detection and identification including DT, ANN, and FDA model. Correct recognition ratio (CRR), area under the receiver operating characteristic curve (ROC), cumulative gains, and lift charts were employed to evaluate the performance of models. According to our results, CRM combined with chemometrics offered a powerful tool for the discrimination of foodborne pathogenic bacteria. As far as we know, this is the first study for the identification of six foodborne pathogenic bacteria using CRM coupled with DT, ANN, and FDA classifiers along with four single pretreatment methods.

## Materials and methods

### Preparation of bacterial samples

The following bacteria were used in the study; they are *Salmonella typhimurium* (*S. typhimurium*) (LT2, Sa 11030), *Shigella flexneri* (*S. flexneri*), *Listeria monocytogenes* (*L. monocytogenes*) (Lin), *Vibrio cholerae* (*V. cholerae*) (Non-toxigenic strain, 93097), *Staphylococcus aureus* (*S. aureus*) (ATCC 25923), and *C. botulinum*. The strains were provided by the State Key Laboratory for Infectious Disease Prevention and Control, National Institute for Communicable Diseases Control and Prevention, and Chinese Center for Disease Control and Prevention (ICDC; Beijing, China).

*C. botulinum* strains were stored in TPGY broth mixed with glycerol at –70^°^C; other strains were housed in Luria-Bertani broth mixed with glycerol at –70^°^C. To multiply bacterial cells, *C. botulinum* strains were grown in TPGY broth for 24 h at 37^°^C anaerobically; other strains were grown in Luria-Bertani broth for 24 h at 37^°^C. The culture media were purchased from Beijing Land Bridge Technology Co., Ltd. (Beijing, China). All spectra were collected during the stationary phase to avoid the influence of different growth periods of bacteria. In this study, their initial and final OD_600_ values are approximately equal based on the results of ultraviolet spectrophotometer (Varian, USA).

### Bacteria sample pretreatment

Each culture was vortexed quickly and centrifuged for 8 min at 4,000 rpm at 4^°^C using Centrifuge 5418 R (Eppendorf, Germany). The supernatant was discarded after centrifugation. Subsequently, 5 mL of 0.9% NaCl solution was added and stirred. This procedure was repeated one time. The sediment was suspended in 1 mL of 0.9% NaCl solution and transferred to a new microcentrifuge tube (1.5 mL). The suspension was centrifuged for 3 min at 13,500 rpm at room temperature using microcentrifuge (Pic017; Heraeus, Germany). This procedure was repeated two times. Finally, the sediment was resuspended in 200 μL of 0.9% NaCl solution. To record the spectra, about 5 μL of solution was dropped onto the aluminum foil and dried at room temperature for 5 min for Raman spectra measuring (Zhang et al., 2021).

### Spectra collection

All Raman experiments were performed using confocal and high performance Raman microscope (XploRA PLUS, HORIBA, Japan). Bacteria samples were detected with a 532-nm laser under a 600 g/mm grating, the laser power is 1 mW, a spectral resolution of 0.6 cm^–1^, and 50X objective lens. The integration time was 10 s. Each bacteria sample was collected six times. Each bacterial spectrum was collected and consisted of 603 points in the range of 600–1,800 cm^–1^. All the spectra were processed *via* Origin software (OriginLab, USA). The background was removed using baseline correction (method: second derivative, baseline mode: user defined, number of baseline points: 16). LabSpec 6.3 (HORIBA, Japan) was utilized to optimize acquisition parameters and collect sufficient Raman spectra for statistical analysis. A single bacteria cell was mapped since the advantage of confocal Raman. Eighty samples of each type of bacteria were cultured, Raman spectra were collected, respectively, and a total of 480 Raman spectra were collected.

By increasing the integration time and the number of scans, the spectra jumper by external interference can be eliminated, and the signal strength of the Raman spectrum can be promoted. However, this method takes longer to collect spectra. Therefore, it is necessary to choose an appropriate integration time and number of scans. Clostridium botulinum was chosen as the test object. Based on the same experimental conditions, the integration time was set to 5, 10, and 15 s, respectively, and the Raman spectra of Clostridium botulinum under different integration times were compared. Based on the same experimental conditions, the number of scans was set to 3, 6, and 9, respectively, and the Raman spectra of Clostridium botulinum under different scan times were compared.

In each bacterium, a sample was randomly selected and tested 10 times in parallel to examine the repeatability of the method.

### Data pretreatment and multivariate analysis

The dataset was preprocessed by four single pretreatment methods, respectively, to explore the impact of various methods on the model. The pretreatment methods included Savitzky–Golay algorithm smoothing (SG), SNV, MSC, and SG 1st Der. Among them, SG can remove the noise of the spectral. SNV and MSC can reduce the influence of scattering on the original spectrum. SG 1st Der can eliminate the interference of the baseline and background. All the preprocessing procedures were carried out in the Unscrambler X 10.4 (CAMO, Norway).

Principal component analysis (PCA) is an unsupervised and multivariate technique that projects a set of correlated features onto a set of uncorrelated features using an orthogonal transformation. It preserves the most of information. In this study, PCA was used for two purposes. The first one is to plot the distribution of data based on the PCA scores to investigate the difference of various samples. The second one is to reduce the dimensionality of the dataset and extract feature. Computational costs of classifiers were reduced, and overfitting was prevented.

Decision tree (DT) gives the various outcomes from a series of decisions based on a flowchart-like diagram. It is a strong tool for planning strategy, research analysis, and making decision. One of primary advantage is easy to follow and understand. There are four popular algorithms in DT model; they are CHAID, exhaustive CHAID, CART, and QUEST algorithm. All of them were applied to build DT models.

ANN is inspired based on modern neuroscience research. The large amount of processing unit is utilized to build a complex model. Human brain neural network structure and function are imitated in the ANN model. ANN is widely applied for spectral analysis and identification (Park and Lek, 2016). There are two popular algorithms in ANN model; they are multilayer perceptron (MLP) and radial basis function (RBF). All of them were applied to build models.

FDA is a supervised technique using a discriminant function to assign data to different groups. FDA is often used to build a model combined with PCA. The PCs from PCA are used in FDA to define and predict classes.

The hold-out method is used as a method of cross-validation in DT, ANN model. In the hold-out method, the dataset is divided into two parts, 70% of the samples are allocated to the training set, and there are 320 samples in the training set. About 30% of the samples are assigned to the testing set, and there are 160 samples in the testing set. They were used to test the predictions of the models (Liu et al., 2018). Leave-one-out cross-validation (LOOCV) technique is used for the cross-validation of FDA model, and it can examine the quality of the classifier and avoid overfitting. In the LOOCV, one spectrum in the dataset is allocated to the validation data and the remaining spectra are allocated to the training data. This process is repeated until each spectrum is used once as the validation data (Tie et al., 2020). Therefore, there are 479 samples in the training set, and there is a sample in the testing set. The tests of equality of group means are used for evaluating the potential of every independent variable before constructing the model. CRR, area under the ROC, cumulative gains, and lift charts were used for comparing the performance of classifiers under different pretreatment methods. All statistic procedures were operated in the IBM SPSS Statistics 25 and the Unscrambler X 10.4 software.

## Results and discussion

The number of scans is closely related to SNR (signal to noise ratio) level of the data, and the scan time is closely associated with Raman intensity of peaks. To acquire the optimal number and time of scans, 3, 6, and 9 scans were tested. About 5, 10, and 15 s for scan time were also tested.

**FIGURE 1:**
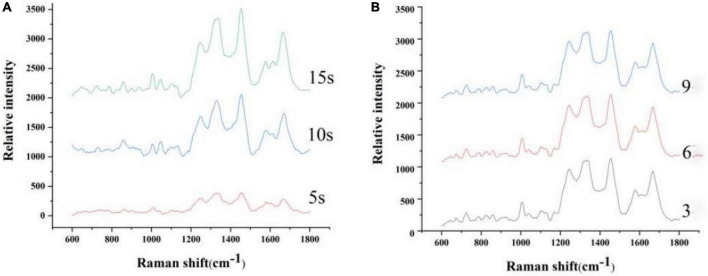
Spectrum based on the different integration times **(A)** and number of scan **(B)**.

Figure 1 is the spectrum based on the different integration times and number of scan. As shown in Figure 1A, the signal intensity of the spectra with an integration time of 10 s is significantly higher than that of 5 s. The peak intensity varied little as the integration time increased. Therefore, 10-s scan time of the sample was considered optimal. As shown in Figure 1B, the peak position and intensity varied little as the number of scan increased. Considering external interfering elements, six scans of the sample were considered optimal.

**FIGURE 2:**
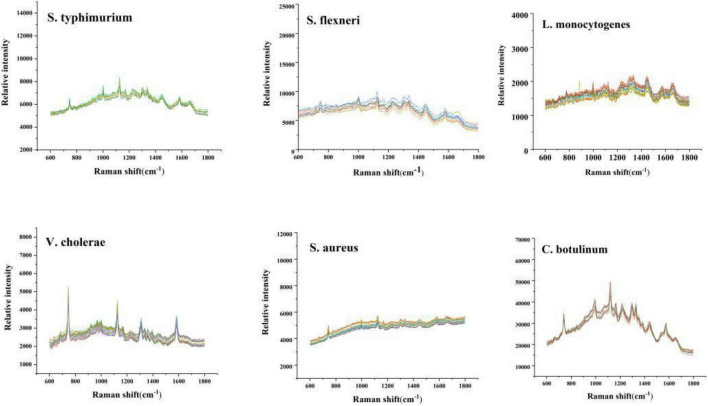
Repetitive experimental trial.

Figure 2 is the repeat experiment in the six kinds of bacteria.

The sample was tested in parallel for 10 times. It can be seen that the spectrum was very similar. The RSD is less than 5%. The result indicated that the repeatability of the experiment is good.

### Visual analysis of spectra

In this study, the CRM technique was used for the detection and identification of six in total foodborne pathogenic bacteria species, namely *S. typhimurium*, *S. flexneri*, *L. monocytogenes*, *V*. *cholerae*, *S. aureus*, and *C*. *botulinum.*

**FIGURE 3:**
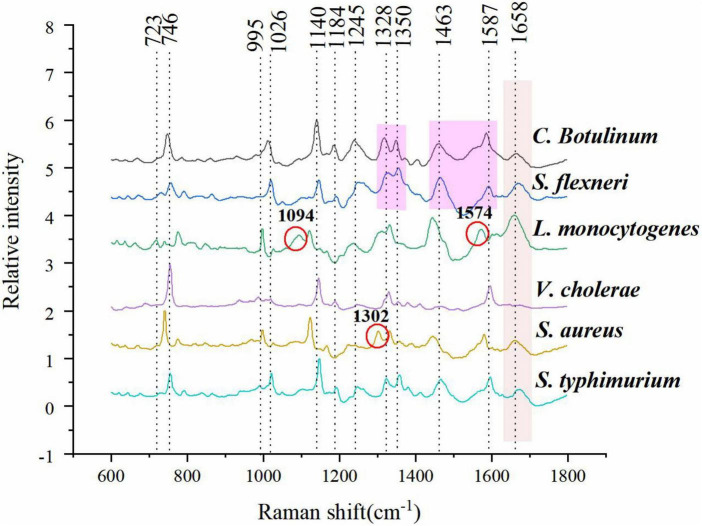
Mean normalized spectra of six bacterial species.

The mean normalized CRM spectra of six bacterial species are shown in Figure 3. Major spectral bands and peaks assignment are shown in Table 1. Some spectral bands are common in all species, and every spectrum showed bands at *ca.* 746, 1,140, and 1,245 cm^–1^ assignable to cytosine and uracil, = C-O-C = (unsaturated fatty acids in lipids), amide III (random), and thymine, respectively. There are also many differences in the spectral images. For example, only *L. monocytogenes* showed a band at ∼1,094 and 1,574 cm^–1^ assignable to CC skeletal and COC stretch (Zheng et al., 2020) from glycosidic link, CN stretching of amide II (Fan et al., 2011), respectively; only *S. aureus* showed a band at ∼1,302 cm^–1^ assignable to amide III (Jarvis et al., 2004). A band was located at 1,658 cm^–1^ in all species except *V*. *cholerae*. There are few differences among *C. botulinum* and *S. flexneri*. However, it is possible to differentiate them by using the ratio of the peak intensities. A total of 1,322/1,350 and 1,463/1,587 were used for differences between *C. botulinum* and *S. flexneri*. A significant difference of peak intensity ratios was shown as depicted in Table 2. Therefore, the different species can be characterized based on the abundant and unique spectral information of CRM.

**TABLE 1:** Raman spectral peak assignment observed in the spectra of six bacterial strains.

Range (cm^–1^)	Assignment (Franco et al., 2017; Witkowska et al., 2017, 2018; Lemma et al., 2019; Uysal et al., 2020)	Range (cm^–1^)	Assignment (Franco et al., 2017; Witkowska et al., 2017, 2018; Lemma et al., 2019; Uysal et al., 2020)
720–740	Adenine	1,213–1,295	Amide III (random), thymine
745–790	Cytosine, uracil	1,320–1,330	Amide III (α-helix)
995–1,010	Phenylalanine, C aromatic ring stretching	1,340–1,365	Amide III (protein)
1,025–1,040	Phenylalanine	1,455–1,465	δ(CH2) wagging
1,120–1,145	= C-O-C= (Unsaturated fatty acids in lipids), galactomannan	1,580–1,590	Guanine ring stretching
1,150–1,195	Aromatic amino acids	1,655–1,670	Amide I (α-helix)

**TABLE 2:** Intensity and intensity ratio of some peaks between *C. botulinum* and *S. flexneri.*

Type	Peak intensity (Arbitr. Units)				Peak intensity ratio	
	1,322 cm^–1^	1,350 cm^–1^	1,463 cm^–1^	1,587 cm^–1^	1,322/1,350	1,463/1,587
*C. Botulinum*	0.29	0.22	0.33	0.28	1.32	1.18
*S. flexneri*	0.07	0.20	0.52	0.23	0.35	2.26

As shown in Figure 3, mean normalized spectra of different bacterial species have a high level of similarity; although they can be differentiated based on several peaks and peak intensity ratio, visual detection of these minor differences is quite time-consuming and may lead to misdiagnosis (Lin et al., 2019). Thus, it is essential to analyze the data combined with multivariate analysis techniques for revealing minor spectral differences. In this study, PCA, DT, ANN, and FDA were used for further study.

### Unsupervised analysis

In this study, the PCA method was used to construct classification models of bacterial species based on the four kinds of pretreatment methods, and it has reduced the dimensionality of the dataset and extracted feature.

**FIGURE 4:**
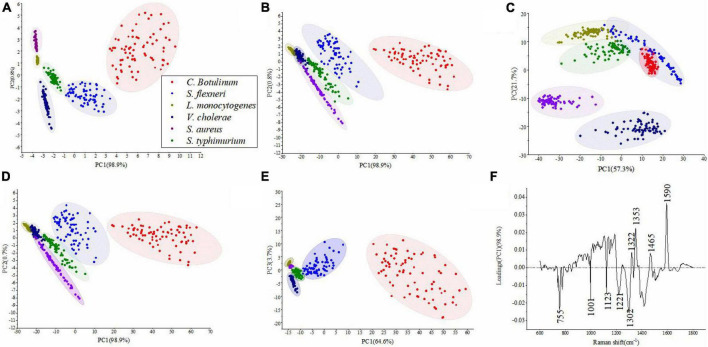
PCA results obtained for six bacterial species. **(A–E)** Represent PCA score plots of various preprocess methods, that is, **(A)** original data, **(B)** SG, **(C)** SNV, **(D)** MSC, and **(E)** SG 1st Der. **(F)** A PC-1 loading plot based on the original data.

Table 3 shows the results of PCA under different pretreatment methods. It can be seen that the number of PCs and corresponding cumulative contribution rate has changed along with difference of pretreatment methods. Figures 4A–E represents PCA score plots under various preprocess methods. These results represented the influence of preprocessed spectra on PCA models and the possibility of differentiation for bacterial species with a high accuracy. Figure 4A shows that all bacterial species can be differentiated. In Figures 4B,D, the samples of *S. aureus* and *S. typhimurium* became more scattered, the categories were closer to each other, and it was difficult to distinguish different categories. In Figure 4C, although the spatial distance of each group became larger, the problem of overlap was more serious. In Figure 4E, the plot had placed samples of the same types closer by SG 1st Der, and overlapping samples were reduced between *C. botulinum* and *S. flexneri* compared to Figure 4C.

**TABLE 3:** Summary of data dimension reduction by PCA under different pretreatment methods.

Pretreatment method	The number of PCs	Cumulative contribution rate (%)
Original data	7	99.97
SG	11	99.99
SNV	4	90.78
MSC	11	99.98
SG 1st Der	6	87.05

Figure 4F represents the most important variables for classification in the original data. The major positive loadings were located at *ca.* 1,322, 1,353, 1,465, and 1,590 cm^–1^. The largest loadings in the positive direction were located at *ca.* 1,353 cm^–1^ (Amide III (protein)) and *ca.* 1,590 cm^–1^ (Guanine ring stretching). The major negative loadings were located at *ca.* 755, 1,001, 1,123, 1,221, and 1,302 cm^–1^. The largest loadings in the negative direction were located at *ca.* 755 cm^–1^ (Cytosine or uracil) and *ca.* 1,302 cm^–1^ (Amide III).

As mentioned above, we can conclude that preliminary classification can be achieved by PCA model combined with the preprocess methods. It is worth noting that PCA is an unsupervised technique, not meant for classification purpose. DT, ANN, and FDA, these supervised and multivariate techniques, were used for the construction of classifiers. They have more advantages than PCA because the information among the classes is taken into account (Wichmann et al., 2020).

To evaluate the potential of every independent variable before constructing the classification model, the tests of equality of group means are used for this study. Supplementary Tables 1–5 give the summary of the tests of equality of group means with spectra preprocessed by various pretreatment methods. In each test, the grouping variable was treated as the factor, and the results of a one-way ANOVA are given for the independent variable. Smaller Wilk’s lambda values suggest that the variable is better at discrimination of groups. If the significance value (Sig) of the independent variable is less than 0.05, it indicates that the variable is significant for the creation of the models. As shown in Supplementary Tables 1–5, Wilk’s lambda value of major PCs is smaller, and every Sig value is far less than 0.5. It indicates that every variable is significant for the construction of the models and there is not any evident anomaly (Chiang et al., 2001).

### Supervised analysis

#### Decision tree model

As depicted in Table 4, comparing with the decision tree based on MSC and SNV, the decision tree based on SG and SG 1st Der had a stronger prediction capacity because of its higher accuracy. SG and SG 1st Der raised accuracy and the ability to extend of DT model compared to original data, and MSC raised accuracy of models in part of algorithms, while SNV resulted in a less model accuracy.

**TABLE 4:** Summary of DT modeling results for bacteria species identification expressed in CRR.

Method	CRR of training set (%)				CRR of testing set (%)			
	CHAID	Exhaustive CHAID	CART	QUEST	CHAID	Exhaustive CHAID	CART	QUEST
Original data	91.9	91.9	90.2	75.7	87.4	87.0	86.7	78.1
SG	91.1	93.2	91.5	84.7	85.8	87.1	89.4	80.3
SNV	88.7	87.4	84.6	83.6	82.2	86.1	84.4	78.7
MSC	90.7	92.5	92.0	85.7	83.7	88.1	89.8	82.6
SG 1st Der	92.2	91.1	100.0	92.3	93.2	91.2	98.1 (157/160)	92.9

The individual performance for the DT method varied according to the species. While some species such as species *S. flexneri*, *L. monocytogenes*, *V*. *cholerae*, and *C*. *botulinum* yielded good prediction performances, some others such as the class *S. aureus* and the *S. typhimurium* fell short from the overall average. The optimal DT model on CRM data was obtained by using CART algorithm (the spectra were preprocessed with SG 1st Der) showing a CRR for training set of 100.0%. On the contrary, the testing set given a CRR of cross-validation of 98.1% (157/160) was correctly classified, one sample from the class S. *aureus* was wrongly allocated to the group *L. monocytogenes*, and two samples from the class S. *typhimurium* were wrongly allocated to the group *S. flexneri* and *V. cholerae*.

**FIGURE 5:**
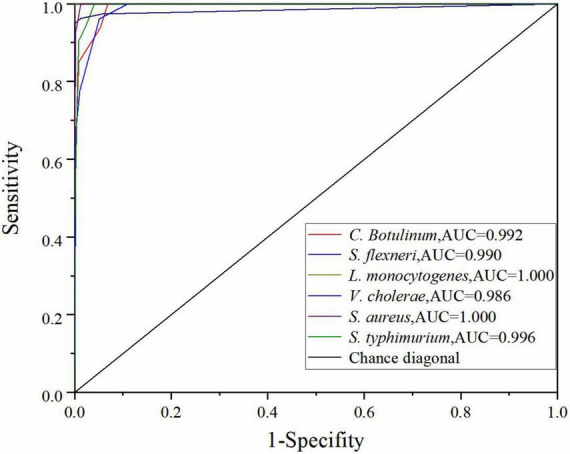
ROC curve of the DT model using the SG 1st Der.

To evaluate the performance of the optimal DT model further, the true positive rate (sensitivity) and true negative rate (specificity) were calculated, and the relationship between sensitivity and specificity was represented by ROC curve graphically for all possible thresholds. The area under the ROC curve (AUC) is widely recognized as the important index of the performance of a model (IBM USA, 2021b). Chance diagonal is a line from (0,0) to (1,1). The area under the chance diagonal is 0.5. The model is effective when AUC is greater than 0.5. As shown in Figure 5, all the AUC values of six bacteria species in the DT model are greater than 0.95. Generally, the AUC value for ROC curve is 1 maximum that indicates that the model is the best classifier and the values closer to 0 indicate poor performance. Thus, the performance of the optimal DT model is excellent (Obuchowski and Bullen, 2018).

#### Artificial neural network model

As shown in Table 5, red-lettered values represent MLP, and blue-lettered values represent RBF. Red-lettered values are higher than the corresponding blue-lettered values; it shows that a model with MLP algorithm has a better performance compared to RBF algorithm, and the ability to find complex relationships is more important in this study. SG and MSC raised accuracy and the ability to extend of MLP model compared to original data. SG 1st Der resulted in the minor reduction in model accuracy, while SNV resulted in a less model accuracy.

**TABLE 5:** Summary of ANN modeling results for bacteria species identification expressed in CRR.

Method	CRR of training set (%)		CRR of testing set (%)	
	MLP	RBF	MLP	RBF
Original data	97.6	87.5	95.6	87.7
SG	99.2	82.7	95.7	75.9
SNV	91.6	91.8	92.1	85.0
MSC	100.0	82.4	99.4 (159/160)	76.0
SG 1st Der	97.2	88.3	94.6	82.2

The individual performance in the ANN model varied according to the species, and some species such as the class *S. flexneri* fell short from the overall average. The optimal ANN model was obtained by using MLP algorithm (the spectra were preprocessed with MSC) showing a CRR for training set of 100.0%. Meanwhile, the testing set provided a CRR of cross-validation of 99.4% (159/160) correctly classified, and one sample from the class *S. flexneri* was wrongly allocated to the group *C. botulinum*. The reason for this can again be attributed to the similarities between the species. However, our collective results do highlight that the DT and ANN models are efficient enough to predict the species identity at the genus level. This is significant since accurate identification of the genus of the foodborne pathogenic bacteria may be sufficient in several applications. For example, in many regulatory practices of food safety, the goal is often to screen a larger sample volume than have detailed characterizations of a very small sample set. In such applications, where fast, efficient, and inter-mediate screening is necessary, perhaps an initial genus-level accuracy is a welcome relief, in which the DT and ANN models are quite capable of providing (Bisgin et al., 2018).

**FIGURE 6:**
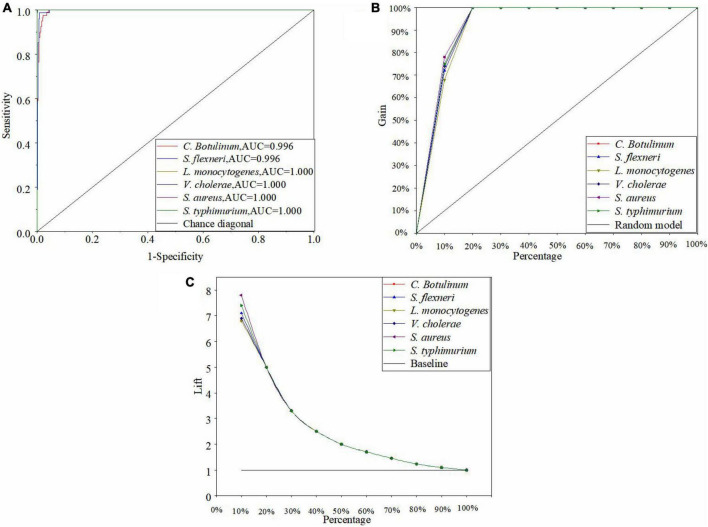
ROC curve **(A)**, cumulative gains **(B)**, and lift charts **(C)** of the optimal ANN model in this study.

Figure 6A is the ROC curve of the ANN model by using the MSC, and all the AUC values of six bacteria species in the ANN model are greater than 0.98. Thus, the performance of the optimal ANN model is superior. Moreover, there are also other powerful methods to assess the performance of the model such as cumulative gains and lift charts. Figure 6B shows the cumulative gain evaluation curve of the optimal ANN model in this study. A lift chart is shown in Figure 6C. The baseline represents the results of random guessing. The lift chart originates directly from the cumulative gain evaluation curve. The X-axis of the lift chart is identical with the cumulative gain evaluation curve, while Y-axis is equal to the ratio of the cumulative gains of independent curve and the baseline. In other word, it presents how many times the model is better than the random choice of samples (IBM USA, 2021a; Nargis et al., 2021). In Figure 6B, every curve rapidly reaches a very high cumulative gain value (100%) and then keeps in level (Tibco USA, 2021). In Figure 6C, each curve is smooth and drops rapidly to 1. The top 10% would contain approximately 70% of the target samples in the ANN model, but it is only 10% without using the model (see Figure 6B). The target samples increase six times with the model (see Figure 6C). As mentioned above, the performance of the optimal ANN model is excellent.

#### Fisher’s discriminant analysis model

The LOOCV technique was used as the cross-validation method to develop FDA model. As shown in the Table 6, the total model accuracy of training set was 93.2% (Original data), 94.0% (SG), 89.3% (SNV), 93.8% (MSC), and 100.0% (SG 1st Der), respectively. The testing set was 92.8% (Original data), 92.8% (SG), 88.9% (SNV), 92.8% (MSC), and 100.0% (SG 1st Der), respectively. The accuracy of training set is similar with the corresponding testing set. It shows that FDA model is fairly robust against overfitting problem. All pretreatment methods contributed in the increase of model accuracy except SNV. The optimal FDA model was obtained by using SG 1st Der algorithm; it resulted in a CRR of training set of 100.0% and a CRR of cross-validation of 100.0%. The individual performance in the FDA model did not vary according to the species, and the different individual species yielded good prediction performances in the FDA model.

**TABLE 6:** Summary of FDA modeling results for bacteria species identification expressed in CRR.

Method	CRR (%)	
	Training set	Testing set
Original data	93.2	92.8
SG	94.0	92.8
SNV	89.3	88.9
MSC	93.8	92.8
SG 1st Der	100.0	100.0

**FIGURE 7:**
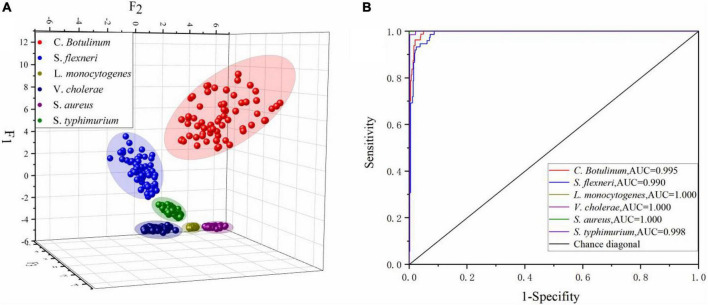
**(A)** All-Groups 3D scatter plot and **(B)** the ROC curve of the FDA model using the SG 1st Der.

Table 7 presents the summary of Fisher’s discriminant functions in the optimal FDA model. In FDA model, Wilk’s lambda values are used to test whether each discriminant function is statistically significant. The range of values is from 0 to 1.0 represents total discrimination, and 1 represents no discrimination, since Wilk’s lambda values of top five functions are greater than 1, and the corresponding Sig is less than 0.05. These five functions are significant in the model. However, Wilk’s lambda value of Function 6 is 1, the corresponding Sig is 0.672 being far greater than 0.05, and variance contribution is 0. Thus, the Function 6 should be discarded. Variance contribution of top three functions is 73.6, 16.7, and 8.5%, respectively. All of these contribute 98.8% of all the variations. All-Groups 3D scatter plot was shown using F_1_, F_2_, and F_3_. As depicted in Figure 7A, six kinds of bacteria species are clearly distinguished between each other with a CRR of 100.0%. Figure 7B represents that all the AUC values of six bacteria species in the DT model are greater than 0.95. In conclusion, our findings demonstrated that CRM and FDA model could be used for the identification of various bacteria species efficiently.

**TABLE 7:** Summary of Fisher’s discriminant functions.

Function	Variance contribution%	Cumulative contribution%	Correlation	Function test	Wilks’ Lambda	Sig
1	73.6	73.6	0.966	1–6	0.005	0.000
2	16.7	90.3	0.872	2–6	0.075	0.000
3	8.5	98.8	0.785	3–6	0.313	0.000
4	0.9	99.7	0.380	4–6	0.815	0.000
5	0.3	100.0	0.218	5–6	0.952	0.000
6	0.0	100.0	0.019	6	1.000	0.672

First five canonical discriminant functions were used in the analysis. F_1_ = 1.126*PC1* + 0.708*PC2* + 0.067*PC3*-0.092*PC4* + 0.050*PC5*-0.125*PC6*. F_2_ = 0.135*PC1*-0.797*PC2* + 0.293*PC3* + 0.987*PC4*-0.240*PC5*-0.188*PC6*. F_3_ = -0.093*PC1* + 0.605*PC2* + 0.789*PC3* + 0.520*PC4* + 0.256*PC5*-0.195*PC6*. F_4_ = 0*PC1* -0.236*PC2* + 0.600*PC3*-0.343*PC4* + 0.283*PC5*-0.240*PC6*. F_5_ = -0.022*PC1*-0.236*PC2* + 0.600*PC3*-0.343*PC4* + 0.283*PC5*-0.240*PC6*.

## Conclusion

The present study evaluates and reveals the potential of the CRM and chemometrics methods for the detection and classification of foodborne pathogenic bacteria. Our findings showed that different bacteria species can be distinguished based on characteristic peaks and peak intensity ratio, but it is time-consuming and not applicable for a large amount of samples. Then, we explored the impact of different pretreatment methods on the models. Most pretreatment methods raised the performance of the models except SNV. Performances of some classical classifiers were compared for bacterial detection and identification. CRR, ROC, cumulative gains, and lift charts were used to evaluate the performance of models. In these studies, preliminary classification can be achieved by PCA model. In the DT and ANN model, there is a difference between the CRR of training set and corresponding testing set. In the FDA model, the CRR of training set is more similar with the corresponding testing set. Therefore, the FDA model is more robust for overfitting problem and offers the highest CRR. In conclusion, CRM and chemometrics offer a powerful tool for the discrimination of foodborne pathogenic bacteria; thus, an application of the technology in the food sector would lead to a huge benefit.

## Data availability statement

The original contributions presented in this study are included in the article/Supplementary material, further inquiries can be directed to the corresponding author/s.

## Author contributions

JZ: methodology, data curation, software, investigation, and writing—original draft. PG: conceptualization and supervision. YW: funding acquisition, supervision, and resources. XY, WL, and MY: supervision and resources. CY: investigation, resources, and supervision. XX: resources, visualization, supervision, funding acquisition, investigation, and editing. HJ: reviewing, validation, resources, and supervision. All authors contributed to the article and approved the submitted version.

## References

[B1] Andrei C. C. Moraillon A. Lau S. Félidj N. Yamakawa N. Bouckaert J. (2020). Rapid and sensitive identification of uropathogenic *Escherichia coli* using a surface-enhanced-Raman-scattering-based biochip. *Talanta* 219:121174. 10.1016/j.talanta.2020.121174 32887096

[B2] Anza I. Skarin H. Vidal D. Lindberg A. Baverud V. Mateo R. (2014). The same clade of *Clostridium botulinum* strains is causing avian botulism in southern and northern Europe. *Anaerobe* 26 20–23. 10.1016/j.anaerobe.2014.01.002 24418766

[B3] Artin I. Mason D. R. Pin C. Schelin J. Peck M. W. Holst E. (2010). Carter, effects of carbon dioxide on growth of proteolytic *Clostridium botulinum*, its ability to produce neurotoxin, and its transcriptome. *Appl. Environ. Microbiol.* 76 1168–1172. 10.1128/AEM.02247-09 20038699 PMC2820955

[B4] Bisgin H. Bera T. Ding H. Semey H. G. Wu L. Liu Z. (2018). Comparing SVM and ANN based machine learning methods for species identification of food contaminating beetles. *Sci. Rep.* 8:6532. 10.1038/s41598-018-24926-7 29695741 PMC5917025

[B5] Braconnier A. Broussolle V. Perelle S. Fach P. Nguyen-The C. Carlin F. (2001). Screening for 250 *Clostridium botulinum* type A, B, and E in cooked chilled foods containing vegetables and raw material using 251 polymerase chain reaction and molecular probes. *J. Food Protect.* 64 201–207. 10.4315/0362-028x-64.2.201 11271768

[B6] Chen L. Alali W. (2018). Editorial: Recent discoveries in human serious foodborne pathogenic bacteria: Resurgence, pathogenesis, and control strategies. *Front. Microbiol.* 9:2412. 10.3389/fmicb.2018.02412 30356687 PMC6189307

[B7] Chiang L. H. Russell E. L. Braatz R. D. (2001). “Tennessee eastman process,” in *Fault detection and diagnosis in industrial systems. Advanced textbooks in control and signal processing* (Bristol: IOP Publishing Ltd).

[B8] Chisanga M. Linton D. Muhamadali H. Ellis D. I. Kimber R. L. Mironov A. (2020). Rapid differentiation of *Campylobacter jejuni* cell wall mutants using Raman spectroscopy, SERS and mass spectrometry combined with chemometrics. *Analyst* 145 1236–1249. 10.1039/c9an02026h 31776524

[B9] Dahlsten E. Korkeala H. Somervuo P. Lindström M. (2008). PCR assay for differentiating between Group I (proteolytic) and Group II (nonproteolytic) strains of *Clostridium botulinum*. *Int. J. Food Microbiol.* 124 108–111. 10.1016/j.ijfoodmicro.2008.02.018 18374440

[B10] De Medici D. Anniballi F. Wyatt G. M. Lindström M. Messelhäusser U. Aldus C. F. (2009). Multiplex PCR for detection of botulinum neurotoxin-producing clostridia in clinical, food, and environmental samples. *Appl. Environ. Microbiol.* 75 6457–6461. 10.1128/AEM.00805-09 19684163 PMC2765140

[B11] Fan C. Hu Z. Mustapha A. Lin M. (2011). Rapid detection of food- and waterborne bacteria using surface-enhanced Raman spectroscopy coupled with silver nanosubstrates. *Appl. Microbiol. Biotechnol.* 92 1053–1061. 10.1007/s00253-011-3634-3 22005743

[B12] Ferreira J. L. Eliasberg S. J. Harrison M. A. Edmonds P. (2001). Detection of preformed type A botulinal toxin in hash brown potatoes by using the mouse bioassay and a modified ELISA test. *J. AOAC Int.* 84 1460–1464. 10.1093/jaoac/84.5.1460 11601465

[B13] Franco D. Trusso S. Fazio E. Allegra A. Musolino C. Speciale A. (2017). Raman spectroscopy differentiates between sensitive and resistant multiple myeloma cell lines. *Spectrochim. Acta A Mol. Biomol. Spectrosc.* 187 15–22. 10.1016/j.saa.2017.06.020 28645097

[B14] Fung F. Wang H. S. Menon S. (2018). Food safety in the 21st century. *Biomed. J.* 41 88–95. 10.1016/j.bj.2018.03.003 29866604 PMC6138766

[B15] Giaouris E. Heir E. Desvaux M. Hébraud M. Møretrø T. Langsrud S. (2015). Intra- and inter-species interactions within biofilms of important foodborne bacterial pathogens. *Front. Microbiol.* 6:841. 10.3389/fmicb.2015.00841 26347727 PMC4542319

[B16] Ho C. S. Jean N. Hogan C. A. Blackmon L. Jeffrey S. S. Holodniy M. (2019). Rapid identification of pathogenic bacteria using Raman spectroscopy and deep learning. *Nat. Commun.* 10:4927. 10.1038/s41467-019-12898-9 31666527 PMC6960993

[B17] Hochel I. Viochna D. Skvor J. Musil M. (2004). Development of an indirect competitive ELISA for detection of *Campylobacter jejuni* subsp.jejuni O:23 in foods. *Folia Microbiol. (Praha)* 49 579–586. 10.1007/BF02931537 15702549

[B18] IBM USA (2021b). *Tests of equality of group means.* Available online at: https://www.ibm.com/support/knowledgecenter/en/SSLVMB_23.0.0/spss/tutorials/discrim_bankloan_groupmean.html (accessed February 22, 2021).

[B19] IBM USA (2021a). *Cumulative gains and lift charts.* Available online at: https://www.ibm.com/support/knowledgecenter/en/SSLVMB_23.0.0/spss/tutorials/mlp_bankloan_outputtype_02.html (accessed February 22, 2021).

[B20] Jarvis R. M. Brooker A. Goodacre R. (2004). Surface-enhanced Raman spectroscopy for bacterial discrimination utilizing a scanning electron microscope with a Raman spectroscopy interface. *Anal. Chem.* 76 5198–5202. 10.1021/ac049663f 15373461

[B21] Kant K. Shahbazi M.-A. Dave V. P. Ngo T. A. Chidambara V. A. Than L. Q. (2018). Microfluidic devices for sample preparation and rapid detection of foodborne pathogens. *Biotechnol. Adv.* 36 1003–1024. 10.1016/j.biotechadv.2018.03.002 29534915

[B22] Keto-Timonen R. Nevas M. Korkeala H. (2005). Efficient DNA fingerprinting of *Clostridium botulinum* types A, B, E, and F by amplified fragment length polymorphism analysis. *Appl. Environ. Microbiol.* 71 1148–1154. 10.1128/AEM.71.3.1148-1154.2005 15746312 PMC1065150

[B23] Kriem L. S. Wright K. Ccahuana-Vasquez R. A. Rupp S. (2020). Confocal Raman microscopy to identify bacteria in oral subgingival biofilm models. *PLoS One* 15:e0232912. 10.1371/journal.pone.0232912 32392236 PMC7213720

[B24] Lemma T. Wang J. Arstila K. Hytönen V. P. Toppari J. J. (2019). Identifying yeasts using surface enhanced Raman spectroscopy. *Spectrochim. Acta Part A Mol. Biomol. Spectrosc.* 218 299–307. 10.1016/j.saa.2019.04.010 31005737

[B25] Lin Z. Zhao X. Huang J. Liu W. Zheng Y. Yang X. (2019). Rapid screening of colistin-resistant *Escherichia coli*, *Acinetobacter baumannii* and *Pseudomonas aeruginosa* by the use of Raman spectroscopy and hierarchical cluster analysis. *Analyst* 144 2803–2810. 10.1039/c8an02220h 30882113

[B26] Lindström M. Keto R. Markkula A. Nevas M. Hielm S. Korkeala H. (2001). Multiplex PCR assay for detection and identification of *Clostridium botulinum* types A, B, E, and F in food and fecal material. *Appl. Environ. Microbiol.* 67 5694–5699. 10.1128/AEM.67.12.5694-5699.2001 11722924 PMC93361

[B27] Liu M. Zhao J. Lu X. Z. Li G. Wu T. Zhang L. (2018). Blood hyperviscosity identification with reflective spectroscopy of tongue tip based on principal component analysis combining artificial neural network. *Biomed. Eng. Online* 17:60. 10.1186/s12938-018-0495-3 29747693 PMC5946417

[B28] Macdonald T. E. Helma C. H. Shou Y. Valdez Y. E. Ticknor L. O. Foley B. T. (2011). Analysis of *Clostridium botulinum* serotype E strains by using multilocus sequence typing, amplified fragment length polymorphism, variable-number tandem-repeat analysis, and botulinum neurotoxin gene sequencing. *Appl. Environ. Microbiol.* 77 8625–8634. 10.1128/AEM.05155-11 22003031 PMC3233090

[B29] Maquelin K. Choo-Smith L. P. Endtz H. P. Bruining H. A. Puppels G. J. (2002). Rapid identification of *Candida* species by confocal Raman microspectroscopy. *J. Clin. Microbiol.* 40 594–600. 10.1128/JCM.40.2.594-600.2002 11825976 PMC153356

[B30] Mi F. Guan M. Hu C. Peng F. Sun S. Wang X. (2021). Application of lectin-based biosensor technology in the detection of foodborne pathogenic bacteria: A review. *Analyst* 146 429–443. 10.1039/d0an01459a 33231246

[B31] Nargis H. F. Nawaz H. Bhatti H. N. Jilani K. Saleem M. (2021). Comparison of surface enhanced Raman spectroscopy and Raman spectroscopy for the detection of breast cancer based on serum samples. *Spectrochim. Acta Part A Mol. Biomol. Spectrosc.* 246:119034. 10.1016/j.saa.2020.119034 33049470

[B32] Ng V. Lin W. J. (2014). Comparison of assembled *Clostridium botulinum* A1 genomes revealed their evolutionary relationship. *Genomics* 103 94–106. 10.1016/j.ygeno.2013.12.003 24369123 PMC3959226

[B33] Obuchowski N. A. Bullen J. A. (2018). Receiver operating characteristic (ROC) curves: Review of methods with applications in diagnostic medicine. *Phys. Med. Biol.* 63:07TR01. 10.1088/1361-6560/aab4b1 29512515

[B34] Olsen J. S. Scholz H. Fillo S. Ramisse V. Lista F. Tromborg A. K. (2014). Analysis of the genetic distribution among members of *Clostridium botulinum* group I using a novel multilocus sequence typing (MLST) assay. *J. Microbiol. Methods* 96 84–91. 10.1016/j.mimet.2013.11.003 24246230

[B35] Park Y. S. Lek S. (2016). “Artificial neural networks: Multilayer perceptron for ecological modeling,” in *Developments in environmental modelling*, Vol. 28 ed. Jorgensen S. E. (Amsterdam: Elsevier), 123–140. 10.1016/B978-0-444-63623-2.00007-4

[B36] Raphael B. H. Joseph L. A. McCroskey L. M. Lúquez C. Maslanka S. E. (2010). Detection and differentiation of *Clostridium botulinum* type A strains using a focused DNA microarray. *Mol. Cell. Probes* 24 146–153. 10.1016/j.mcp.2009.12.003 20056143

[B37] Serrano P. Hermelink A. Lasch P. de Vera J. P. König N. Burckhardt O. (2015). Confocal raman microspectroscopy reveals a convergence of the chemical composition in methanogenic archaea from a siberian permafrost-affected soil. *FEMS Microbiol. Ecol.* 91:fiv126. 10.1093/femsec/fiv126 26499486

[B38] Skarin H. Lindberg A. Blomqvist G. Aspán A. Båverud V. (2010). Molecular characterization and comparison of *Clostridium botulinum* type C avian strains. *Avian Pathology* 39 511–518. 10.1080/03079457.2010.526923 21154062

[B39] Szabo E. A. Pemberton J. M. Gibson A. M. Eyles M. J. Desmarchellier P. M. (1994). Polymerase chain reaction for the detection of *Clostridium botulinum* types A, Band E in food, soil and infant faeces. *J. Appl. Bacteriol.* 76 539–545. 10.1111/j.1365-2672.1994.tb01650.x 8027003

[B40] Taban B. M. Ben U. Aytac S. A. (2009). Rapid detection of *Salmonella* in milk by combined immunomagnetic separation-polymerase chain reaction assay. *J. Dairy Sci.* 92 2382–2388. 10.3168/jds.2008-1537 19447970

[B41] Tibco USA, (2021). *Gains vs ROC curves. Do you understand the difference?* Available online at: https://community.tibco.com/wiki/gains-vs-roc-curves-do-you-understand-difference (accessed February 22, 2021).

[B42] Tie Y. Duchateau C. Van de Steene S. Mees C. De Braekeleer K. De Beer T. (2020). Spectroscopic techniques combined with chemometrics for fast on-site characterization of suspected illegal antimicrobials. *Talanta* 217:121026. 10.1016/j.talanta.2020.121026 32498874

[B43] Umeda K. Seto Y. Kohda T. Mukamoto M. Kozaki S. (2009). Genetic Characterization of *Clostridium botulinum* associated with Type B infant botulism in Japan. *J. Clin. Microbiol.* 47 2720–2728. 10.1128/JCM.00077-09 19571018 PMC2738102

[B44] Uysal C. F. Saridag A. M. Kilic I. H. Tokmakci M. Kahraman M. Aydin O. (2020). Identification of methicillin-resistant *Staphylococcus aureus* bacteria using surface-enhanced Raman spectroscopy and machine learning techniques. *Analyst* 145, 7559–7570. 10.1039/d0an00476f 33135033

[B45] Vanhomwegen J. Berthet N. Mazuet C. Guigon G. Vallaeys T. Stamboliyska R. (2013). Application of high-density DNA resequencing microarray for detection and characterization of botulinum neurotoxin-producing clostridia. *PLoS One* 8:67510. 10.1371/journal.pone.0067510 23818983 PMC3688605

[B46] Vaz-Velho M. Duarte G. Gibbs P. (2000). Evaluation of mini-VIDAS rapid test for detection of *Listeria monocytogenes* from production lines of fresh to cold-smoked fish. *J. Microbiol. Methods* 40 147–151. 10.1016/s0167-7012(00)00118-4 10699670

[B47] Wang X. Slavik M. F. (1999). Rapid detection of *Salmonella* in chicken washes by immunomagnetic separation and flow cytometry. *J. Food Protect.* 62:717. 10.4315/0362-028x-62.7.717 10419261

[B48] Wichmann C. Bocklitz T. Rösch P. (2020). *Spectrochim. Acta A Mol. Biomol. Spectrosc.* 248:119170. 10.1016/j.saa.2020.119170 33296748

[B49] Witkowska E. Jagielski T. Kamińska A. (2017). Genus- and species-level identification of dermatophyte fungi by surface-enhanced Raman spectroscopy. *Spectrochim. Acta A Mol. Biomol. Spectrosc.* 192 285–290. 10.1016/j.saa.2017.11.008 29156315

[B50] Witkowska E. Jagielski T. Kamińska A. (2018). Genus- and species-level identification of dermatophyte fungi by surface-enhanced Raman spectroscopy. *Spectrochim. Acta A Mol. Biomol. Spectrosc.* 192 285–290. 10.1016/j.saa.2017.11.008 29156315

[B51] Wu X. Xu C. Tripp R. A. Huang Y. W. Zhao Y. (2013). Detection and differentiation of foodborne pathogenic bacteria in mung bean sprouts using field deployable label-free SERS devices. *Analyst* 138, 3005–3012. 10.1039/c3an00186e 23563168

[B52] Yan B. Cheng A. C. Wang M. S. Deng S. X. Zhang Z. H. Yin N. C. (2008). Application of an indirect immunofluorescent staining method for detection of *Salmonella enteritidis* in paraffin slices and antigen location in infected duck tissues. *World J. Gastroenterol.* 14 776–781. 10.3748/wjg.14.776 18205271 PMC2684008

[B53] Yin M. Jing C. Li H. Deng Q. Wang S. (2020). Surface chemistry modified upconversion nanoparticles as fluorescent sensor array for discrimination of foodborne pathogenic bacteria. *J. Nanobiotechnol.* 18:41. 10.1186/s12951-020-00596-4 32111217 PMC7049179

[B54] Zhang J. Jiang H. Gao P. Wu Y. Sun H. Huang Y. (2021). Confocal Raman microspectroscopy combined with chemometrics as a discrimination method of clostridia and serotypes of *Clostridium botulinum* strains. *J. Raman Spectrosc.* 52, 1820–1829. 10.1002/jrs.6244

[B55] Zheng X. Wu G. Lv G. Yin L. Luo B. Lv X. (2021). Combining derivative Raman with autofluorescence to improve the diagnosis performance of echinococcosis. *Spectrochim. Acta A Mol. Biomol. Spectrosc.* 247:119083. 10.1016/j.saa.2020.119083 33137629

[B56] Zhou C. Xu W. Zhang P. Jiang M. Chen Y. Kwok R. T. K. (2019). Engineering sensor arrays using aggregation-induced emission luminogens for pathogen identification. *Adv. Funct. Mater.* 29 1805986.1–1805986.10. 10.1039/d2an00643j 35611940

