# Supplementary material for: Identification of foodborne pathogenic bacteria using confocal Raman microspectroscopy and chemometrics
Source: Front Microbiol. 2022 Nov 7;13:874658. doi: 10.3389/fmicb.2022.874658 (PMC9676656; doi:10.3389/fmicb.2022.874658)
Supplement: Supplementary file 1 [file Data_Sheet_1.doc]

**Supplementary Materials**

**Tab S1.**

The tests of equality of group means on original spectra

| Variable | Wilks’ Lambda | F | df1 | df2 | Sig |
| --- | --- | --- | --- | --- | --- |
| PC1 | 0.091 | 867.777 | 6 | 524 | 0.000 |
| PC2 | 0.409 | 126.309 | 6 | 524 | 0.000 |
| PC3 | 0.514 | 82.609 | 6 | 524 | 0.000 |
| PC4 | 0.836 | 17.138 | 6 | 524 | 0.000 |
| PC5 | 0.909 | 8.751 | 6 | 524 | 0.000 |
| PC6 | 0.515 | 82.334 | 6 | 524 | 0.000 |
| PC7 | 0.733 | 31.807 | 6 | 524 | 0.000 |

**Tab S2.**

The tests of equality of group means on spectra preprocessed by SG

| Variable | Wilks’ Lambda | F | df1 | df2 | Sig |
| --- | --- | --- | --- | --- | --- |
| PC1 | 0.091 | 867.767 | 6 | 524 | 0.000 |
| PC2 | 0.409 | 126.255 | 6 | 524 | 0.000 |
| PC3 | 0.518 | 81.222 | 6 | 524 | 0.000 |
| PC4 | 0.834 | 17.326 | 6 | 524 | 0.000 |
| PC5 | 0.906 | 9.101 | 6 | 524 | 0.000 |
| PC6 | 0.533 | 76.389 | 6 | 524 | 0.000 |
| PC7 | 0.736 | 31.333 | 6 | 524 | 0.000 |
| PC8 | 0.984 | 1.408 | 6 | 524 | 0.000 |
| PC9 | 0.752 | 28.751 | 6 | 524 | 0.000 |
| PC10 | 0.959 | 3.706 | 6 | 524 | 0.001 |
| PC11 | 0.763 | 27.093 | 6 | 524 | 0.000 |

**Tab S3.**

The tests of equality of group means on spectra preprocessed by SNV

| Variable | Wilks’ Lambda | F | df1 | df2 | Sig |
| --- | --- | --- | --- | --- | --- |
| PC1 | 0.170 | 426.974 | 6 | 524 | 0.000 |
| PC2 | 0.092 | 860.325 | 6 | 524 | 0.000 |
| PC3 | 0.251 | 261.171 | 6 | 524 | 0.000 |
| PC4 | 0.165 | 442.881 | 6 | 524 | 0.000 |

**Tab S4.**

The tests of equality of group means on spectra preprocessed by MSC

| Variable | Wilks’ Lambda | F | df1 | df2 | Sig |
| --- | --- | --- | --- | --- | --- |
| PC1 | 0.091 | 867.777 | 6 | 524 | 0.000 |
| PC2 | 0.409 | 126.309 | 6 | 524 | 0.000 |
| PC3 | 0.514 | 82.609 | 6 | 524 | 0.000 |
| PC4 | 0.836 | 17.138 | 6 | 524 | 0.000 |
| PC5 | 0.909 | 8.751 | 6 | 524 | 0.000 |
| PC6 | 0.515 | 82.334 | 6 | 524 | 0.000 |
| PC7 | 0.733 | 31.807 | 6 | 524 | 0.000 |
| PC8 | 0.984 | 1.404 | 6 | 524 | 0.000 |
| PC9 | 0.755 | 28.342 | 6 | 524 | 0.000 |
| PC10 | 0.955 | 4.109 | 6 | 524 | 0.000 |
| PC11 | 0.773 | 25.615 | 6 | 524 | 0.000 |

**Tab S5.**

The tests of equality of group means on spectra preprocessed by SG 1st Der

| Variable | Wilks’ Lambda | F | df1 | df2 | Sig |
| --- | --- | --- | --- | --- | --- |
| PC1 | 0.093 | 849.093 | 6 | 524 | 0.000 |
| PC2 | 0.383 | 140.504 | 6 | 524 | 0.000 |
| PC3 | 0.730 | 32.245 | 6 | 524 | 0.000 |
| PC4 | 0.463 | 101.483 | 6 | 524 | 0.000 |
| PC5 | 0.874 | 12.540 | 6 | 524 | 0.000 |
| PC6 | 0.954 | 4.178 | 6 | 524 | 0.000 |





**Fig S1.** Importance of every PC in the ANN model preprocessed with the MSC.
